# How to motivate residents’ behavioral intention to engage in household waste sorting? An integrated perspective based on the technology acceptance model and the theory of planned behavior

**DOI:** 10.3389/fpsyg.2026.1817955

**Published:** 2026-05-13

**Authors:** Jianwei Cao, Haoxiang Sun, Min Ding

**Affiliations:** 1 School of Business, North Minzu University, Yinchuan, China; 2 School of Marxism, North Minzu University, Yinchuan, China

**Keywords:** behavioral intention, residents, technology acceptance model, theory of planned behavior, waste sorting

## Abstract

Waste sorting at the source represents the starting point of waste classification management and directly affects the effectiveness of subsequent classified collection, transportation, and treatment. As the primary actors in household waste sorting, residents play a critical role in the successful implementation of household waste classification management. Therefore, motivating residents’ behavioral intention to engage in household waste sorting is essential for improving the overall effectiveness of waste classification management. From an integrated perspective of the technology acceptance model (TAM) and the theory of planned behavior (TPB), this study developed a theoretical model to explain the formation mechanism of residents’ behavioral intention to engage in household waste sorting. Using a random sampling approach, a questionnaire survey was conducted among Chinese residents, yielding 635 valid responses. The results indicated that perceived usefulness and perceived ease of use of household waste sorting positively influence behavioral intention; attitude, subjective norms, and perceived behavioral control of household waste sorting significantly and positively predict behavioral intention; attitude of household waste sorting fully mediates the relationship between perceived usefulness and behavioral intention; moreover, both attitude and perceived behavioral control of household waste sorting partially mediate the relationship between perceived ease of use and behavioral intention. These findings provide valuable insights into strategies for motivating residents’ behavioral intention to participate in household waste sorting.

## 1 Introduction

In recent years, with the acceleration of global urbanization, the volume of household waste generated has continued to increase. Traditional waste management practices characterized by “mixed collection and centralized landfilling” not only occupy substantial land resources but also tend to cause soil, water, and air pollution, posing serious threats to ecological sustainability and public health. Against this backdrop, household waste sorting has become an important issue in environmental protection and resource utilization worldwide. Within the process of household waste classification management, waste sorting at the source constitutes the starting point and directly determines the effectiveness of subsequent classified collection, transportation, and treatment. Source-based waste sorting can significantly improve the efficiency of downstream classification processes and increase the recovery rate of recyclable resources, thereby enhancing lifecycle carbon emission reduction benefits (Gundupalli et al., 2017). Conversely, inaccurate sorting at the disposal stage increases the cost and difficulty of waste classification management and may even lead to its failure. Consequently, household waste sorting at the source has emerged as a key strategy for addressing the challenge of “cities besieged by waste” and for achieving waste reduction, resource recovery, and harmless disposal.

Household waste sorting at the source is a complex social system project involving multiple stakeholders, including governments, enterprises, communities, and residents. Among these actors, residents play a fundamental and decisive role in the waste classification chain, as they are the primary generators of household waste and the direct executors of waste sorting at the source. The standardization and continuity of residents’ waste sorting behaviors directly determine whether household waste classification management can be effectively implemented. Furthermore, behavioral intention (hereinafter BI) serves as a prerequisite for actual behavior, only when residents possess a strong BI to engage in household waste sorting are they more likely to translate such BI into actual sorting behavior. Residents’ BI to engage in household waste sorting is measured by individuals’ subjective assessment of the likelihood that they will perform household waste sorting behaviors. That is, the extent to which they are willing to sort household waste. Motivating residents’ BI to engage in household waste sorting represents both a focal point and a major challenge in waste classification management. In practice, many residents perceive waste sorting as inconvenient or troublesome (Howenstine, 1993) and continue to follow previous unsorted disposal habits, lack of residents’ BI has become a major bottleneck hindering the efficient operation of waste classification systems (Dai et al., 2025). Therefore, a systematic examination of the influencing factors and underlying mechanisms of residents’ BI is of considerable theoretical and practical significance for designing effective incentive strategies and enhancing the effectiveness and sustainability of waste sorting behaviors.

Existing studies indicate that residents’ waste sorting BI is jointly influenced by multiple factors. These factors include external contextual influences at the social level, such as moral norms (Li et al., 2023), policies and regulations (Xu et al., 2017), publicity and education (Wang et al., 2019), the adequacy of sorting facilities (Tonglet et al., 2004; Kim, 2023), and information intervention (Li et al., 2024), as well as individual-level psychological and cognitive factors, such as awareness of the importance of waste sorting (Park and Ha, 2014; Wu et al., 2022), personal norms (Tang et al., 2023), emotional contagion (Gu et al., 2025), and sense of belonging (Zhang et al., 2025). Nevertheless, most existing studies focus on isolated or limited sets of influencing factors and lack a theoretical framework capable of systematically integrating multidimensional determinants while clearly explaining the formation pathways of residents’ waste sorting BI. This fragmented research landscape makes it difficult to capture the complexity of BI formation comprehensively and limits the precision of practical intervention strategies. Accordingly, there is an urgent need to identify and integrate key determinants that explain residents’ BI to engage in household waste sorting and to systematically investigate the underlying formation mechanisms.

The Technology Acceptance Model (hereinafter TAM), proposed by Davis (1989) and derived from the Theory of Reasoned Action, aims to explain and predict individuals’ acceptance of technology. The core premise of TAM is that individuals’ acceptance intentions are jointly determined by perceived usefulness (hereinafter PU) and perceived ease of use (hereinafter PEOU) (Davis, 1989). PU refers to the extent to which an individual believes that using a particular technology will enhance task or performance outcomes, whereas PEOU reflects the extent to which an individual believes that using the technology requires minimal effort. Although TAM was initially developed within the research stream of information technology adoption, its underlying theoretical mechanism is not confined to information technology itself; rather, it emphasizes how individuals’ perceptions of the use of external technology support are formed and, subsequently, translated into behavioral intentions. When individuals perceive higher usefulness and ease of use, their BI to perform the corresponding behavior becomes stronger. Owing to its parsimony and explanatory power, TAM has been widely applied in research on explaining individual intentions and behaviors (Kim et al., 2009; Marangunic and Granic, 2015; Sohn and Kwon, 2020). Specifically, in the context of residents’ household waste sorting and recycling services, residents’ intention to participate depends closely on their perceptions of whether the external sorting and disposal support technology is “useful” and “easy to use”(Wildt and Meijers, 2023; Zhang et al., 2024; Nakao and Seo, 2026). Residents’ perceptions of whether waste sorting support technology contributes to environmental protection and resource recycling, as well as whether the sorting process is simple and convenient, may significantly influence their BI to engage in such behavior. Information guidance that clarifies sorting procedures, disposal rules, and potential benefits can help reinforce residents’ perceptions of whether waste sorting is PU and PEOU. Therefore, when household waste sorting relies substantially on external sorting technology support, applying TAM to explain residents’ participation intentions is contextually transferable.

The Theory of Planned Behavior (hereinafter TPB), developed by Ajzen (1991) based on the Theory of Reasoned Action, is a central framework in social psychology for explaining and predicting human behavior. TPB posits that BI is the most immediate antecedent of actual behavior and that intention is determined by three core constructs: attitude toward the behavior (hereinafter ATB), subjective norm (hereinafter SN), and perceived behavioral control (hereinafter PBC) (Ajzen, 1991). ATB refers to an individual’s positive or negative evaluation of performing a specific behavior; SN reflects perceived social pressure, that is, whether important others (e.g., family members, friends, or community neighbors) expect the individual to perform the behavior and whether the individual is motivated to comply with such expectations; and PBC denotes the perceived ease or difficulty of performing the behavior, reflecting confidence in possessing the necessary resources, opportunities, and abilities. When individuals hold favorable attitudes toward behavior, perceive strong social expectations, and believe they have sufficient control over performing the behavior, their BI tends to be stronger. TPB emphasizes the role of cognitive and volitional factors in behavioral decision-making and has been shown to substantially enhance the explanatory and predictive power of behavioral research (Steinmetz et al., 2016; Yuriev et al., 2020). Therefore, applying TPB to the context of household waste sorting recognizes that residents’ BI is shaped by their psychological cognition, including their attitudes toward waste sorting, perceived social norms, and perceived control over sorting behaviors.

As discussed above, a substantial body of research suggests that residents typically weigh multiple factors—both external environmental influences and internal psychological cognition—when deciding whether to engage in household waste sorting. TAM explains the formation of BI from the perspective of external technological support, focusing on residents’ PU and PEOU regarding waste sorting, whereas TPB explains intention formation from the perspective of individual psychological cognition through ATB, SN, and PBC. Given the limitations of relying on a single theory to explain complex social behaviors, this study integrates TAM and TPB to examine the formation process of residents’ household waste sorting behavior. Specifically, PU and PEOU in TAM provide concrete external antecedents for key TPB constructs such as ATB and PBC, while ATB, SN, and PBC in TPB compensate for TAM’s relative neglect of individual psychological cognition. This integrative perspective enables the combination of external technological considerations (PU and PEOU) with internal psychological factors (ATB, SN, and PBC), thereby constructing a more comprehensive analytical framework to systematically and deeply elucidate the formation mechanisms of residents’ BI to engage in household waste sorting.

Accordingly, this study integrated TAM and TPB to construct a theoretical model explaining the formation mechanism of residents’ BI to engage in household waste sorting. The study aimed to examine the direct effects of PU, PEOU, ATB, SN, and PBC on residents’ BI and to test the potential mediating roles of ATB in the relationships between PU and BI and between PEOU and BI, as well as the mediating role of PBC in the relationship between PEOU and BI. To this end, data were collected from residents in China using a random sampling approach, yielding 635 valid responses for quantitative analysis. Empirical tests indicated that all proposed hypotheses were supported, and the theoretical model demonstrated strong validity. The contributions of this study can be summarized in three aspects.

First, based on TAM, this study treats household waste sorting as residents’ acceptance of an external technological support and identifies PEOU and PU as two salient external technology support perceptions that shape residents’ behavioral intention. Prior studies have extensively examined the waste-recycling domain using TAM, demonstrating that cognitive variables such as perceived usefulness and perceived ease of use can effectively account for residents’ acceptance and participation intentions in recycling contexts (Wildt and Meijers, 2023; Zhang et al., 2024; Nakao and Seo, 2026). In particular, while prior TAM-oriented waste-sorting studies have suggested that PU and PEOU can relate to intention outcomes in related domains (Zhang et al., 2024; Nakao and Seo, 2026), our findings go a step further by specifying the TAM-consistent mechanism through which these external technology support perceptions operate in this behavioral setting. By bringing TAM’s core logic into the waste-sorting context, our theorization helps expand the relatively limited TAM-based research in household waste-sorting settings and provides a more mechanism-oriented explanation of how residents’ perceptions translate into intention (Davis, 1989; Marangunic and Granic, 2015; Sohn and Kwon, 2020; Zhang et al., 2024).

Second, based on TPB, this study centers on residents’ internal cognition and clarifies that ATB, SN, and PBC represent three core cognitive pathways through which residents form waste-sorting intention. Existing TPB-based studies examining the formation of behavioral intention often operationalize these constructs in a relatively fragmented manner (Zhang et al., 2019; Bardus and Massoud, 2022), thereby resulting in insufficient consistency in their theoretical explanations. By further conceptual clarification and refinement of ATB, SN, and PBC, our findings enrich TPB’s application in the household waste-sorting context and provide a more mechanism-consistent account of how intention is cognitively constructed, in line with the broader TPB literature emphasizing the joint roles of evaluative, normative, and control beliefs in shaping intention (Steinmetz et al., 2016; Yuriev et al., 2020). Notably, using evidence from a China-based dataset, we further demonstrate that these TPB-based cognitive pathways remain meaningful across varying levels of exposure to waste-sorting initiatives and across differing institutional contexts. Compared with prior TPB applications, the overall logic of our framework strengthens TPB’s context-sensitive explanatory power for waste-sorting intention and pointing toward a more structured, cognition-focused direction for future TPB research in this domain (Xu et al., 2017; Wang et al., 2021).

Third, from an integrated TAM-TPB perspective, this study systematically revealed the mechanism underlying residents’ BI to engage in household waste sorting. Current research in this area often adopts single-model explanations or provides fragmented mediation evidence (Yuriev et al., 2020; Zhang et al., 2024; Nakao and Seo, 2026), which means that the mechanism through which external technology support perceptions are translated into the internal intention-formation process remains insufficiently explained. This study advances an integrated TAM-TPB explanation by uncovering three mediation mechanisms that clarify how external technology-environment perceptions translate into intention through internal cognitions. Specifically, ATB fully mediates the PU → BI relationship, both ATB and PBC partially mediate the PEOU→BI relationship. These findings provide a more fine-grained account of the intention-formation process by explicating the pathways through which PU and PEOU exert influence, thereby addressing the theoretical integration lack and fragmented conclusions in prior waste-sorting intention research (Kuang and Lin, 2021; Li et al., 2023; Cao et al., 2024; Liang et al., 2024) and echoing the need for more mechanism-oriented, integrative frameworks in this domain (Xu et al., 2018; Zhang et al., 2024).

The remainder of this article is structured as follows. First, the research hypotheses and theoretical model are presented. Second, the measurement of variables and data collection procedures are described. Third, the empirical analysis results are reported. Finally, the findings are discussed, managerial implications are derived, and the study’s limitations and directions for future research are outlined.

## 2 Research hypotheses and theoretical model

### 2.1 Main effects of PU and PEOU on BI

According to the TAM, household waste sorting depends on external technological support perceptions through which the determinants of residents’ BI to engage in household waste sorting can be examined. BI of household waste sorting results from the combined effects of PU and PEOU. PU of household waste sorting refers to the extent to which residents perceive that engaging in waste sorting technological support leads to positive outcomes, such as reducing environmental pollution, improving resource recovery rates, and enhancing the quality of the household residential environment. When residents perceive household waste sorting technological support as highly useful, they are more likely to regard this behavior as valuable and worth the effort, thereby strengthening their belief in participation and increasing their BI to engage in waste sorting. Conversely, if residents perceive that household waste sorting technological support contributes little to environmental improvement or resource recycling, their motivation and BI to participate in waste sorting are likely to be suppressed. PEOU of household waste sorting reflects residents’ perceptions of the convenience and effort required to waste sorting technological support, including the comprehensibility of sorting standards, the time and effort required for sorting, and the availability and convenience of sorting facilities. When residents perceive the waste sorting technological support as simple and easy to understand, requiring minimal time and effort, and supported by accessible and convenient facilities, the barriers to participation are reduced, thereby increasing their BI to engage in waste sorting. In contrast, when residents perceive sorting technological support as complex, such as procedures as cumbersome, or facilities as inconvenient, they may develop resistance toward waste sorting, which in turn diminishes their BI. Accordingly, when residents perceive household waste sorting technological support as PU (“recognizing” its value) and PEOU (“finding” it easy), they are more likely to develop stronger BI (“wanting” to do) to engage in household waste sorting.

Based on these arguments, the following hypotheses are proposed:

H1: Residents’ PU of household waste sorting positively influences their BI.

H2: Residents’ PEOU of household waste sorting positively influences their BI.

### 2.2 Main effects of ATB, SN and PBC on BI

According to the TPB, residents’ BI to engage in household waste sorting can be attributed to individual psychological cognition, further elucidating the determinants of BI. Residents’ BI of household waste sorting is jointly determined by their ATB, SN, and PBC.

ATB reflects residents’ positive or negative evaluations of engaging in waste sorting behavior. When residents hold positive feelings toward waste sorting and perceive it as necessary, they are more likely to develop favorable attitudes and stronger BI to engage in waste sorting. Conversely, when residents hold negative feelings and perceive waste sorting as unnecessary, they are likely to adopt unfavorable attitudes and exhibit lower BI to participate. SN refers to the social pressure residents perceive regarding whether they should engage in household waste sorting. When residents perceive stronger social pressure—namely, clearer expectations or constraints from significant others or society—they tend to experience stronger subjective norms, which in turn enhance their BI to engage in waste sorting. In contrast, when perceived social pressure is weak and expectations are unclear, SN diminish, leading to reduced BI. PBC represents residents’ overall assessment of their past experiences and anticipated obstacles related to household waste sorting. When residents perceive that they possess sufficient skills, external support, and resources, and anticipate fewer difficulties in performing waste sorting, their PBC becomes stronger, thereby facilitating the activation of BI. Conversely, when residents perceive insufficient skills or support and anticipate greater difficulties, their PBC weakens, making it more difficult to motivate their BI. Therefore, when residents hold positive ATB of household waste sorting (“should” do), perceive strong SN (“must” do), and experience high PEC (“can” do), they are more likely to develop stronger BI (“want” to do) to engage in household waste sorting.

Based on these considerations, the following hypotheses are proposed:

H3: Residents’ ATB of household waste sorting positively influences their BI.

H4: Residents’ SN of household waste sorting positively influences their BI.

H5: Residents’ PBC of household waste sorting positively influences their BI.

### 2.3 Mediation effects of ATB and PBC

The TPB further suggests that attitude and perceived behavioral control are influenced by behavioral beliefs and control beliefs, respectively (Ajzen, 1991). In this context, PU in TAM constitutes a key component of behavioral beliefs, whereas PU and PEOU jointly form important components of control beliefs (Davis, 1989). Residents’ PU of household waste sorting, namely, the extent to which they believe that waste sorting technological support leads to outcomes such as environmental improvement and resource recovery, directly shapes their evaluation of the behavior and subsequently influences whether they form positive or negative ATB toward waste sorting. When residents strongly recognize the usefulness of waste sorting technological support, they are more likely to develop positive ATB; conversely, when PU is low, negative ATB may emerge. Residents’ ATB of household waste sorting then exert a spillover effect on their BI, such that positive ATB further strengthen their BI, whereas negative ATB weaken it. Accordingly, PU can indirectly motivate residents’ BI to engage in household waste sorting by enhancing their ATB. Residents’ PEOU of household waste sorting reflecting perceptions of operational simplicity, facility convenience, and time efficiency directly influences both their ATB toward waste sorting and their PBC. When residents perceive waste sorting technological support as easy and requiring minimal effort, they are more likely to develop positive ATB and stronger PBC. In contrast, when the waste sorting technological support is perceived as complex or burdensome, residents may experience resistance, leading to negative ATB and lower PBC. ATB and PBC, in turn, exert spillover effects on their BI, such that positive ATB and stronger PBC further enhance their BI. Therefore, PEOU can indirectly motivate residents’ BI to engage in household waste sorting by improving their ATB and PBC.

Based on the above analysis, the following hypotheses are proposed:

H6: Residents’ ATB of household waste sorting mediates the relationship between their PU and BI.

H7: Residents’ ATB of household waste sorting mediates the relationship between their PEOU and BI.

H8: Residents’ PBC of household waste sorting mediates the relationship between their PEOU and BI.

### 2.4 Theoretical model

**Figure 1:**
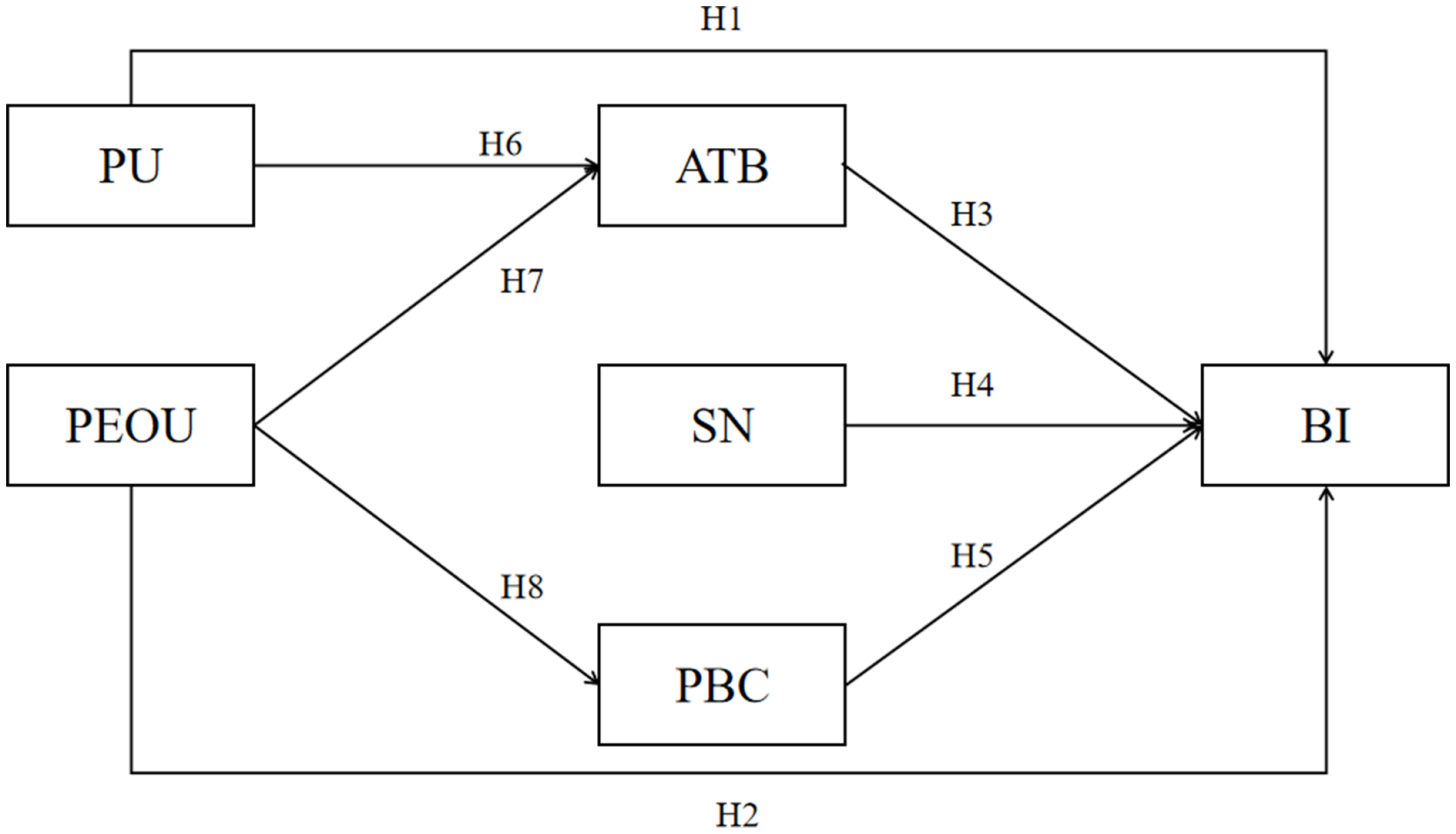
The model for motivating residents’ BI.

Based on the proposed hypotheses, this study further constructs a theoretical model of the formation mechanism of residents’ BI to engage in household waste sorting, as illustrated in Figure 1.

## 3 Research design

### 3.1 Measurement of variables

In applying the TAM and the TPB to examine the formation mechanism of residents’ BI to engage in household waste sorting, this study adapts established measurement instruments to the specific research context of household waste sorting among Chinese residents. Widely used and well-validated scales from prior research were appropriately modified to reflect the concrete practices and cultural context of household waste sorting in China. Specifically, the measurement items for PU and PEOU of household waste sorting were adapted from the technology acceptance scales originally developed by Davis (1989). The measurement items for ATB, SN, PBC, and BI of household waste sorting were adapted from the scales developed by Elliott et al. (2003), which have been extensively used to measure BI, ATB, SN, and PBC. The scale adaptation process respected the conceptual meanings of each construct while incorporating the cultural and situational characteristics of household waste sorting in China. Prior to formal data collection, to ensure the scientific rigor and contextual applicability of the measurement instruments, several experts in environmental behavior and social survey research were invited to review and repeatedly revise the wording accuracy, logical coherence, and cultural appropriateness of the scale items. Through this iterative refinement process, the final version of the questionnaire was established, effectively ensuring content validity and enabling accurate measurement of Chinese residents’ genuine perceptions and attitudes toward household waste sorting. All core variables were measured using a five-point Likert scale ranging from 1 (“strongly disagree”) to 5 (“strongly agree”). Higher scores indicate a higher level of agreement with the corresponding statements. All measures were based on residents’ self-reported responses. The specific measurement items for each construct are presented in Table 1.

**Table 1:** Measurement instruments for variables.

Variable	Measurement items
Behavioral intention (BI)	BI1. I am willing to consistently sort household waste in my daily life. BI2. I will proactively learn relevant knowledge and methods of household waste sorting to perform it more effectively. BI3. Even without strict supervision, I am willing to voluntarily engage in household waste sorting.
Perceived usefulness (PU)	PU1. Household waste sorting with supporting facilities is beneficial for environmental protection. PU2. Standardized household waste sorting helps promote resource recycling and reuse. PU3. Improving the intelligent level of waste sorting facilities exerts a positive effect on optimizing the living environment.
Perceived ease of use (PEOU)	PEOU1. The operational procedures of household waste sorting are simple and easy for me to master. PEOU2. The labels on household waste sorting containers are clear and help me complete accurate sorting with ease. PEOU3. Engaging in household waste sorting in daily life does not cause me much additional inconvenience.
Attitude towards the behavior (ATB)	ATB1. I believe that household waste sorting is a practice worth promoting. ATB2. I hold a supportive attitude toward household waste sorting behavior. ATB3. In my view, actively participating in household waste sorting is a civic responsibility. ATB4. Overall, I have a positive evaluation of household waste sorting behavior.
Subjective norm (SN)	SN1. People around me (such as family members, neighbors, and colleagues) expect me to engage in household waste sorting. SN2. If I do not engage in household waste sorting, I may receive negative evaluations from people around me. SN3. Most people in society believe that household waste sorting is something that should be done. SN4. Publicity and advocacy by communities or relevant authorities regarding household waste sorting make me feel that it is necessary to do so.
Perceived behavioral control (PBC)	PBC1. I am confident that I can persist in household waste sorting over the long term. PBC2. Even when sorting standards are occasionally adjusted, I can adapt quickly and sort waste correctly. PBC3. In daily life, I am able to successfully overcome minor difficulties encountered during household waste sorting. PBC4. I am confident in my ability to correctly complete household waste sorting.

Previous studies indicate that individuals’ demographic characteristics can influence BI (Zhang et al., 2019; Wang et al., 2021; Li et al., 2023; Kan et al., 2025). Accordingly, to control for the potential effects of individual characteristics on the hypothesis testing results, this study incorporates a set of demographic variables as control variables. Specifically, eight demographic variables were included: gender (male, female); age (20 years or younger, 21–40 years, 41 years or older); educational attainment (secondary school or vocational school and below, college or university, postgraduate); annual household income (below RMB 100,000, RMB 100,000–300,000, above RMB 300,000); place of residence (urban, rural); Implement: whether the community has implemented household waste sorting programs (yes, no); Organization: whether the community organizes household waste sorting activities (yes, no); Requirement: whether the community requires mandatory household waste sorting (yes, no). All demographic information was self-reported by the residents at the time of the survey.

### 3.2 Data collection

In January 2026, we conducted an online paid questionnaire survey using the Wenjuanxing platform. As one of the largest survey platforms in China, Wenjuanxing is widely used for various types of questionnaire data collection. The target population of this study was residents in general rather than a specific subgroup, our survey was administered through Wenjuanxing’s participant pool. Specifically, Wenjuanxing applied a random sampling-based invitation procedure within its pool: eligible panel members who met the study’s participation requirements were randomly invited to access and complete the questionnaire, whereas open voluntary participation (e.g., self-selection through public links) was not used. To further improve the representativeness of the achieved sample, we requested the platform to monitor the demographic composition of incoming responses during recruitment, and we conducted a post-recruitment composition check to evaluate whether the final sample was broadly consistent with the general demographic characteristics of Chinese residents. Participation was anonymous, and the survey introduction clearly stated the academic purpose and data-use policy. Respondents who fill out the questionnaire carefully will receive a certain amount of electronic cash rewards. in order to ensure response quality. A total of 680 questionnaires were collected. After excluding responses with invalid response patterns, 635 valid responses were retained for subsequent analyses (valid responses among collected questionnaires = 93.3%). Specifically, we excluded invalid responses based on the following criteria: (1) respondents were required to pass two embedded attention-check questions, these attention-check items were designed to identify respondents who completed the survey without reading items carefully, respondents who failed these checks were considered ineligible for inclusion in the final dataset; (2) completing the survey in less than one-third of the median completion time; and (3) providing clearly invalid responses characterized by inconsistent and non-sensical patterns across related items (e.g., contradictory answers that indicate careless or random responding). These predefined exclusion thresholds were applied consistently across all collected responses.

Among the 635 valid responses, 279 respondents were male (43.9%) and 356 were female (56.1%), indicating a relatively higher proportion of female participants. In terms of age, 53 respondents (8.3%) were aged 20 years or younger, 500 respondents (78.7%) were aged 21–40 years, and 82 respondents (12.9%) were aged 41 years or older. Regarding educational attainment, 40 respondents (6.3%) had completed secondary school or vocational education or below, 508 respondents (80.0%) held a college or university degree, and 87 respondents (13.7%) held a postgraduate degree or above. With respect to annual household income, 150 respondents (23.6%) reported an income below RMB 100,000, 424 respondents (66.8%) reported an income between RMB 100,000 and RMB 300,000, and 61 respondents (9.6%) reported an income above RMB 300,000. Regarding place of residence, 534 respondents (85.5%) lived in urban areas, while 92 respondents (14.5%) lived in rural areas, indicating a relatively higher proportion of urban residents. In addition, 519 respondents (81.7%) reported that their communities had implemented household waste sorting programs; 426 respondents (67.1%) indicated that their communities organized household waste sorting activities; and 380 respondents (59.8%) reported that their communities required mandatory household waste sorting. We performed another composition check by comparing key demographic distributions in the final sample (e.g., gender, age, education, income, and urban/rural residence) with known population characteristics of Chinese residents. The retained sample distributions were found to be broadly comparable. Overall, the achieved sample obtained via platform-based randomized invitation is broadly consistent with the general characteristics of the Chinese resident population, supporting the plausibility of using this sample to investigate residents’ behavioral intention toward household waste sorting in the Chinese context.

## 4 Data analysis and empirical results

### 4.1 Descriptive statistics, correlations, reliability, and validity analysis

The results of the descriptive statistics, correlation analysis, and reliability and validity tests are presented in Table 2. With respect to descriptive statistics, the mean values of the six core variables are relatively high (with the lowest mean being 3.872), while the standard deviations are relatively small (with the highest value being 0.725). These results indicate that Chinese residents generally exhibit strong BI, PU, PEOU, SN, ATB, and PBC toward household waste sorting, with relatively low internal variability among respondents. Regarding correlations, all six core variables are positively correlated with one another, suggesting strong associations among BI, PU, PEOU, SN, ATB, and PBC. Moreover, all correlation coefficients are below 0.70, providing preliminary evidence that multicollinearity is not a serious concern in this study. In terms of reliability and validity, the Cronbach’s *α* coefficients of all six core variables exceed 0.60 (with the lowest value being 0.635), meeting the commonly accepted threshold and indicating satisfactory internal consistency reliability. The average variance extracted (AVE) values for all constructs are greater than 0.50 (with the lowest value being 0.569), and the composite reliability (CR) values all exceed 0.80 (with the lowest value being 0.805), falling within acceptable ranges and demonstrating good convergent validity. Furthermore, the square roots of the AVE values for each construct are greater than the corresponding inter-construct correlation coefficients, indicating adequate discriminant validity among the variables.

**Table 2:** descriptive statistics, correlations, and reliability and validity analysis.

Variables	Mean	SD	BI	PU	PEOU	SN	ATB	PBC	Cronbach’α	AVE	CR
BI	4.059	0.688	**(0.816)**						0.749	0.667	0.857
PU	4.392	0.549	0.459**	**(0.761)**					0.635	0.579	0.805
PEOU	3.921	0.765	0.633**	0.341**	**(0.811)**				0.739	0.657	0.852
SN	3.872	0.725	0.619**	0.392**	0.649**	**(0.770)**			0.768	0.592	0.852
ATB	4.345	0.549	0.608**	0.698**	0.510**	0.546**	**(0.754)**		0.746	0.569	0.841
PBC	3.989	0.709	0.664**	0.418**	0.684**	0.699**	0.607**	**(0.786)**	0.794	0.618	0.866

** indicates significance at the 0.01 level; * indicates significance at the 0.05 level. ** bold values on the diagonal represent the square roots of the AVE.

### 4.2 Common method Bias test and confirmatory factor analysis

Due to the data in this study were collected using residents’ self-reported questionnaires, it is necessary to examine the potential presence of common method variance (CMV). Harman’s single-factor test was employed to assess common method bias by loading the six core variables onto a single factor. The results show that the first principal component accounts for 40.01% of the total variance, which is below the commonly accepted threshold of 50%. This indicates that common method bias is not a serious concern in the present study. The results of the confirmatory factor analysis (CFA) are reported in Table 3. The six-factor model demonstrates the best overall fit to the data, with all fit indices meeting recommended criteria (χ^2^ = 461.337, df = 155, RMSEA = 0.052, χ^2^/df = 2.976, SRMR = 0.049, CFI = 0.932, TLI = 0.916). These results indicate that the theoretical model constructed in this study is empirically sound and acceptable. In contrast, the single-factor model exhibits the poorest fit, with none of the fit indices meeting the recommended thresholds. This further confirms that the constructs exhibit good discriminant validity and that serious common method bias is unlikely to be present. The results of the reliability analysis are presented in Table 2. As shown, the Cronbach’s *α* coefficients for all six core constructs exceeded 0.7, which is higher than the conventional threshold of 0.6. This indicates that the measurement scales used in this study demonstrate good internal consistency and high reliability.

**Table 3:** Confirmatory factor analysis results.

Model	χ^**2**^	df	χ^**2**^**/**df	CFI	TLI	RMSEA	SRMR
Six-Factor Model (PU; PEOU; BI; ATB; SN; PBC)	461.337	155	2.976	0.932	0.916	0.052	0.049
Five-Factor Model (PU + PEOU; BI; ATB; SN; PBC)	669.752	160	4.185	0.867	0.842	0.071	0.063
Four-Factor Model (PU + PEOU; BI+ATB; SN; PBC)	697.948	164	4.255	0.860	0.838	0.072	0.064
Three-Factor Model (PU + PEOU+SN; BI+ATB; PBC)	715.178	167	4.282	0.857	0.837	0.072	0.063
Two-Factor Model (PU + PEOU+SN + BI+ATB; PBC)	708.640	169	4.193	0.859	0.841	0.071	0.062
Single-Factor Model (PU + PEOU+BI+ATB + SN + PBC)	725.859	170	4.269	0.855	0.838	0.072	0.063

### 4.3 Regression analysis

#### 4.3.1 Main effects tests

The results of the main effects tests are reported in Models 1–5 of Table 4. According to the regression results of Model 1, PU has a significant positive effect on BI (*β* = 0.543, *p* < 0.01), providing support for H1. The regression results of Model 2 indicate that PEOU has a significant positive effect on BI (*β* = 0.516, *p* < 0.01), supporting H2. As shown in Model 3, ATB significantly and positively influences BI (*β* = 0.718, *p* < 0.01), thus supporting H3. The results of Model 4 demonstrate that SN has a significant positive effect on BI (*β* = 0.527, *p* < 0.01), providing support for H4. Finally, the regression results of Model 5 show that PBC exerts a significant positive effect on BI (*β* = 0.588, *p* < 0.01), thereby supporting H5. In summary, the regression results from Models 1 through 5 consistently verify that perceived usefulness, perceived ease of use, attitude toward the behavior, subjective norm, and perceived behavioral control all have significant positive effects on residents’ BI to engage in household waste sorting. Accordingly, Hypotheses H1–H5 are fully supported.

**Table 4:** Analysis results of main, mediation, and moderation effects.

Variables	Main effect					Mediation effect					
	Model 1	Model 2	Model 3	Model 4	Model 5	Model 6	Model 7	Model 8	Model 9	Model 10	Model 11
	BI	BI	BI	BI	BI	ATB	BI	ATB	BI	PBC	BI
Cons	1.152**	1.827**	0.431	1.801**	1.568**	1.267**	0.322	2.957**	0.364	1.27**	1.344**
Gender	0.033	0.038	0.001	0.022	−0.008	0.052	−0.001	0.078*	−0.001	0.098*	0.001
Age	0.048	0.056	0.054	0.046	0.029	−0.002	0.05	0.031	0.041	0.069	0.03
Education	−0.023	−0.031	0.004	−0.013	−0.034	−0.038	0.002	−0.044	−0.01	0.009	−0.034
Income	−0.011	−0.04	−0.001	−0.017	−0.039	−0.015	−0.001	−0.045	−0.018	0.006	−0.043
Place	0.032	−0.063	0.039	−0.03	0.038	−0.008	0.037	−0.072	−0.028	−0.115	−0.019
Implement	0.109	0.073	0.074	0.053	0.023	0.049	0.077	0.022	0.062	0.105	0.033
Organization	0.177**	0.101	0.157**	0.115*	0.116*	0.034	0.155*	0.008	0.097	0.045	0.084
Requirement	0.328**	0.203**	0.307**	0.105	0.171**	0.029	0.309**	−0.063	0.234**	0.121*	0.157**
PU	0.543**					0.691**	0.091				
PEOU		0.516**						0.376**	0.330**	0.596**	0.289**
SN				0.527**							
ATB			0.718**				0.655**		0.495**		
PBC					0.588**						0.380**
R^2^	0.336	0.448	0.471	0.405	0.472	0.496	0.474	0.274	0.562	0.500	0.525
R^2^ad	0.326	0.440	0.464	0.396	0.464	0.488	0.465	0.263	0.555	0.492	0.518
F	35.12**	56.45**	61.89**	47.24**	62.02**	68.25**	56.21**	26.16**	80.03**	69.32**	69.01**

**indicates significance at the 0.01 level; * indicates significance at the 0.05 level.

#### 4.3.2 Mediation effects tests

The results of the mediation effect tests are reported in Models 6–11 of Table 4. According to the regression results of Models 6 and 7, PU has a significant positive effect on ATB (*β* = 0.691, *p* < 0.01). After controlling for ATB, the effect of PU on BI becomes nonsignificant (*β* = 0.091, *p* > 0.05), indicating that ATB fully mediates the relationship between PU and BI. Therefore, H6 is supported. Based on the regression results of Models 8 and 9, PEOU has a significant positive effect on ATB (*β* = 0.376, *p* < 0.01). After controlling for ATB, the effect of PEOU on BI remains positive and significant (*β* = 0.330, *p* < 0.01), suggesting that ATB plays a partial mediating role in the relationship between PEOU and BI. Thus, H7 is supported. According to the regression results of Models 10 and 11, PEOU has a significant positive effect on PBC (*β* = 0.596, *p* < 0.01). After controlling for PBC, the effect of PEOU on BI remains positive and significant (*β* = 0.289, *p* < 0.01), indicating that PBC partially mediates the relationship between PEOU and BI. Therefore, H8 is supported.

To further examine the statistical significance of the mediating effects of ATB and PBC, a bootstrap analysis was conducted using Mplus 8.3, with 10,000 resamples and a 95% confidence interval. The results are presented in Table 5. The mediating effect of ATB between PU and BI is 0.396, with a 95% confidence interval of [0.339, 0.454], which does not include zero. Meanwhile, after controlling for ATB, the direct effect of PU on BI (c’) becomes nonsignificant, with a 95% confidence interval of [−0.100, 0.190], which includes zero. According to the mediation testing criterion (i.e., a*b is significant and c’ is nonsignificant), the above results indicate that ATB plays a “full mediation” role in the relationship between PU and BI, providing additional support for H6. It should be noted that although c’ is not statistically significant, its point estimate is not exactly zero (the direct effect is approximately *β* = 0.091). Therefore, we report the proportion mediated as 81.5%, that is [0.396/(0.396 + 0.090)], indicating that the mediated share is <100%. This suggests that, at the sample level, there may still be a very small direct path effect. However, because the corresponding confidence interval crosses zero, this direct effect cannot be statistically confirmed under the current data and is thus still classified as “full mediation.” The mediating effect of ATB between PEOU and BI is 0.300, with a 95% confidence interval of [0.241, 0.359], which does not include zero. Meanwhile, after controlling for ATB, the direct effect of PEOU on BI remains significant (*β* = 0.330), with a 95% confidence interval of [0.273, 0.386]. These findings indicate that ATB partially mediates the relationship between PEOU and BI, further supporting H7. The corresponding proportion mediated for PEOU→ATB → BI is 47.6%, that is 0.300 / (0.300 + 0.330). Similarly, the mediating effect of PBC between PEOU and BI is 0.390, with a 95% confidence interval of [0.326, 0.453], which does not include zero. After controlling for PBC, the direct effect of PEOU on BI remains significant (*β* = 0.289), with a 95% confidence interval of [0.221, 0.357]. These results demonstrate that PBC plays a significant partial mediating role in the relationship between PEOU and BI, further supporting H8. The proportion mediated for PEOU→PBC → BI is 57.4%, that is 0.390 / (0.390 + 0.289). Overall, the bootstrap results are consistent with the stepwise regression findings reported in Table 4. Accordingly, Hypotheses H6–H8 are all supported.

**Table 5:** Bootstrap confidence interval results for mediation effects.

Path	Effect	SE	95% CI (Lower)	95% CI (Upper)	Mediation type
Mediation Path 1: PU—ATB—BI					
PU—ATB (a1)	0.691	0.030	0.642	0.740	
ATB—BI (b1)	0.573	0.039	0.510	0.637	
Indirect effect (a1*b1)	0.396	0.035	0.339	0.454	
PU—BI (c1’)	0.090	0.051	−0.100	0.190	Full Mediation
Mediation Path 2: PEOU—ATB—BI					
PEOU—ATB (a2)	0.523	0.038	0.460	0.586	
ATB—BI (b2)	0.574	0.039	0.510	0.637	
Indirect effect (a2*b2)	0.300	0.036	0.241	0.359	
PEOU—BI (c2’)	0.330	0.029	0.273	0.386	Partial Mediation
Mediation Path 3: PEOU—PBC—BI					
PEOU—ATB (a3)	0.644	0.031	0.592	0.695	
ATB—BI (b3)	0.605	0.044	0.533	0.678	
Indirect effect (a3*b3)	0.390	0.039	0.326	0.453	
PEOU—BI (c3’)	0.289	0.034	0.221	0.357	Partial Mediation

c1’ represents the effect of PU on BI after controlling for ATB; c2’ represents the effect of PEOU on BI after controlling for ATB; and c3 represents the effect of PEOU on BI after controlling for PBC.

## 5 Conclusions and discussion

### 5.1 Conclusion

Household waste sorting is a critical yet challenging component of waste classification management. As residents are the primary generators of household waste and the direct executors of waste sorting behaviors, motivating residents’ BI to engage in household waste sorting is a key prerequisite for ensuring the effectiveness of waste classification policies. At present, waste sorting management in many countries and regions around the world remains in an exploratory stage, and scientifically grounded and effective approaches to motivating residents’ participation in household waste sorting are still lacking. Consequently, theoretical research must respond to practical needs by systematically uncovering the formation mechanisms underlying residents’ BI of household waste sorting, thereby providing actionable guidance for addressing challenges in waste classification management. Based on the integrated TAM-TPB framework, this study constructed a theoretical model to explain the formation mechanism of residents’ BI of household waste sorting. Using a random sampling approach, questionnaire data were collected from Chinese residents, yielding 635 valid responses. Descriptive analysis, correlation analysis, reliability analysis, confirmatory factor analysis, and regression analysis were conducted using SPSS and Mplus. The empirical results indicated that PU and PEOU significantly and positively influenced BI of household waste sorting; ATB, SN, and PBC also exerted significant positive effects on BI. Furthermore, ATB fully mediated the relationship between PU and BI, while ATB and PBC partially mediated the relationship between PEOU and BI. By integrating TAM and TPB for the first time in the context of household waste sorting, this study jointly considers external technological support conditions and individual cognitive psychological factors, systematically examining multiple formation pathways and mediating mechanisms of residents’ BI. These findings provide a more comprehensive theoretical perspective for understanding residents’ decision-making processes regarding household waste sorting behavior.

### 5.2 Managerial implications

Drawing on evidence from the large-sample analysis, this study derives three interrelated managerial implications for strengthening residents’ BI to participate in household waste sorting.

First, strengthen external technological support and fully leverage the “technology system” to improve residents’ PU and PEOU. To enhance PU, community managers should shift from advocacy-oriented communication to performance-driven governance, making the value of waste sorting tangible. Specifically, they should reinforce residents’ judgments through quantified feedback on governance outcomes. For example, link sorting performance to measurable indicators such as improvements in recycling efficiency, tighter control of disposal costs, and enhanced neighborhood sanitation. A closed-loop communication mechanism—“indicators—actions—outcomes”—can be implemented through monthly/quarterly governance dashboards that report recyclables recovery rates, reductions in mixed disposal, and the timeliness of collection and removal. This approach reduces information asymmetry and increases policy credibility. In addition, performance-based operating rules should be institutionalized, so that coverage of collection points, responsiveness to mixed-disposal correction, and service timeliness are built into assessment and evaluation. As a result, residents can consistently observe in daily life that sorting produces better real-world outcomes. To improve PEOU, technological design and system usability should be optimized in parallel, lowering residents’ effort costs through better site placement and streamlined procedures. Conduct situational assessments for collection-point placement—e.g., considering building entrances, residents’ typical walking routes, and high-frequency communal disposal locations. In areas prone to frequent mis-sorting, adjust bin placement and nearby prompts accordingly. At the interface level, standardize clear, concise graphic and text labels and simplify decision cues so that “what to sort / what not to sort” is displayed directly at the disposal openings. Where necessary, provide multilingual, plain-language guidance. For categories that are often mis-sorted (e.g., recyclable packaging disposed of incorrectly), implement localized bin configuration adjustments and dynamic correction strategies to reduce the cost of error recovery. Finally, update signage based on mis-sorting data to keep usability practical and sustainable.

Second, focus on residents’ internal cognition and consolidate behavioral drivers under the TPB framework. Because residents’ intention to sort waste depends on three cognitive pathways—ATB, SN, and PBC—interventions should map directly onto “attitude shaping—norm reinforcement—capability support.” Concretely, to strengthen ATB, communities should provide value-oriented, targeted messaging and demonstrations that residents can easily perceive. This can include establishing locally credible positive exemplars —e.g., model households exhibiting civic behavior, and deploying recognition and incentive mechanisms that highlight both the social significance and the practical benefits of waste sorting, thereby improving residents’ positive evaluations of behavioral outcomes. To reinforce SN, use collective commitments, institutionalized norm communication, and demonstrations led by authoritative community figures to stabilize social expectations and shared identification. For example, organize commitment activities, disclose overall compliance progress in a timely and transparent manner, and coordinate with property management and community backbone members so that normative signals remain consistent and sustainable. To enhance PBC, translate residents’ “whether they will do it” and “whether they can do it successfully” concerns into actionable support systems. This can be achieved through simplified, context-specific explanations of sorting rules, on-site guidance during the initial rollout phase, and convenient consultation and feedback channels —e.g., service counters, hotlines, and volunteer assistance—to reduce operational uncertainty and behavioral friction.

Third, implement an integrated intervention strategy based on mechanism coupling to achieve a linked transformation from “technology support—cognitive formation—intention generation.” Drawing on the study’s integrated mediation framework, management should deliver targeted interventions along the three mechanism pathways to ensure that technological improvements translate into residents’ behavioral intention in a stable and systematic way. For PU, the public performance of waste sorting should be translated into personal benefits that residents can understand, compare, and connect to everyday well-being. These benefits should be reinforced through timely and verifiable performance feedback, consolidating the “worthwhile” basis of attitudes and forming a stable evaluative orientation (effective, beneficial, and worth adopting). This, in turn, promotes ATB and drives BI —e.g., regularly publicizing recycling outcomes, reductions in mixed disposal, and environmental improvements, while linking feedback to subsequent governance actions. For PEOU, construct an intervention chain of “easy-to-do experience—positive evaluation—internalized attitude.” By streamlining procedures, providing step-by-step guidance, and using error-tolerant prompts, reduce residents’ learning and decision-making burden. This helps ensure that, in real practice, residents experience sorting as easy and results as predictable, thereby strengthening their positive evaluations of behavioral value. Ultimately, ATB supports the translation into BI. Meanwhile, usability improvements should also support the development of PBC by ensuring that residents have both “initially usable” and “continuously usable” means to carry out sorting. Error correction and resource provision should be institutionalized—for example, by offering rapid on-site/online consultation, responsive correction for mis-sorting, and timely clarification and guidance for difficult cases. This reduces uncertainty and the risk of failure, helping residents form the belief that “I can do it and I can do it successfully,” thereby enabling a more robust transmission from PBC to BI. Overall, implement mechanism-targeted pilots in representative communities, and periodically monitor changes in PU/PEOU, ATB, PBC, and BI, as well as the magnitude of pathway effects. Based on the results, identify and standardize the intervention combinations that show the most stable effects within each pathway, and then scale them up for broader implementation. In this way, the technology system (PU/PEOU) can be translated continuously and stably into residents’ waste-sorting intentions through the mediating mechanisms (ATB and PBC).

### 5.3 Limitations and future research directions

Theoretical models require continuous testing and refinement through empirical application, and the formation mechanism model proposed in this study likewise warrants further validation across broader contexts and longer time horizons. First, limitations related to the empirical sample should be acknowledged. Although 635 valid questionnaires were obtained through simple random sampling approach, the sample was confined to a single national context—China. As a result, the findings may not be directly generalizable to countries or regions with different institutional arrangements, social norms, and cultural value orientations. Future studies may expand the geographical scope of sampling and examine the applicability of the proposed model using cross-national data. Second, we acknowledge limitations stemming from the data-collection procedure and the overall study design. Although the survey used a randomized recruitment strategy through the Wenjuanxing participant pool and the resulting data passed common quality checks (e.g., reliability/validity and common method bias diagnostics), the dataset was obtained from a single round of cross-sectional, self-reported questionnaires, does not capture temporal dynamics among PU/PEOU, internal cognitions (ATB, SN, and PBC), and BI. Therefore, even with randomized recruitment, the measured perceptions may not perfectly reflect residents’ stable waste-sorting behaviors, and residual biases such as recall inaccuracies and social desirability effects may still affect the estimated relationships among the constructs. Future studies should therefore consider longitudinal or multi-wave designs to better examine change over time and strengthen causal interpretation. In addition, experimental or quasi-experimental approaches (e.g., policy or intervention trials) could be used to test whether the proposed mechanisms operate as intended. Furthermore, to improve measurement credibility, future research may incorporate objective or third-party indicators (e.g., community sorting records or audit-based observations) to complement self-reports and provide convergent evidence. Third, the extensibility of the theoretical model can be further enhanced. Based on the integrated TAM-TPB framework, this study focuses on five core antecedents—PU, PEOU, ATB, SN, and PBC, to explain residents’ BI of household waste sorting. Future research could incorporate additional variables and control variables, such as moral obligation (Schwartz, 1977), environmental values (Werff et al., 2013), and policy trust (Levi and Laura, 2000), as potential mediators or moderators. In particular, we suggest incorporating external contextual factors—such as relevant environmental policies and enforcement intensity—into the discussion or future research model, since these “opportunity-structure” elements may directly shape residents’ perceptions and behavioral intentions. Doing so would better reflect the interactive relationship between institutional and infrastructural environments and individual psychological factors. It also helps fully unpack the complex mechanisms driving residents’ behavioral intention toward household waste sorting.

## Data Availability

The original contributions presented in the study are included in the article/supplementary material, further inquiries can be directed to the corresponding author.
